# Complementary Methods in Cancer Treatment—Cure or Curse?

**DOI:** 10.3390/ijerph18010356

**Published:** 2021-01-05

**Authors:** Kaja Michalczyk, Jakub Pawlik, Izabela Czekawy, Mateusz Kozłowski, Aneta Cymbaluk-Płoska

**Affiliations:** Department of Gynecological Surgery and Oncology of Adults and Adolescents, Pomeranian Medical University, al. Powstańców Wielkopolskich 72, 70-111 Szczecin, Poland; jakubpawlik13@gmail.com (J.P.); i.czekawy@gmail.com (I.C.); mtkoozo@gmail.com (M.K.); anetac@data.pl (A.C.-P.)

**Keywords:** complementary medicine, alternative treatment, chemotherapy, cancer

## Abstract

(1) Background: The prevalence of complementary and alternative methods (CAM) use among oncological patients has been rising constantly over the last few decades and a variety of both pharmacological and non-pharmacological methods have been developed. Many advertisements promise to relieve side effects of chemotherapy or even to cure the disease, thus encouraging patients to use CAM; (2) Methods: The objective of the study was to determine which patients’ characteristics are associated with the use of complementary medicine during cancer treatment, their pattern of use, and if it has any association with its safety profile. This survey-based prospective multicenter study of 316 patients examined the use of complementary medicine among patients undergoing chemotherapy treatment in cancer centers in Poland between 2017 and 2019; (3) Results: The Chi2 analysis showed that patients’ opinion regarding the safety of unconventional methods is related to the use of CAM (*p* = 0.00147). Moreover, patients’ thinking that alternative medicine can replace traditional therapy was correlated with his/her education (*p* = 0.01198). Moreover, we performed univariate and multivariate analysis to determine factors associated with CAM use including sociodemographic and clinical characteristics. Finally, we conducted survival analysis of patients undergoing chemotherapy treatment with 42 months of follow-up time of our prospective study. Using Kaplan–Meier curves and log-rank analysis, we found no statistical difference in overall survival between the groups that used and did not use any form of CAM (*p* = 0.211); (4) Conclusions: CAM use is common among patients undergoing chemotherapy treatment and should be considered by medical teams as some agents may interact with chemotherapy drugs and affect their efficacy or cause adverse effects.

## 1. Introduction

After having poor prognosis or multiple adverse effects from chemotherapy, many patients seek alternative methods to cure cancer. Even though current methods include local (surgery or radiotherapy), target, and systematic treatment, we are still unable to help some patients, especially those diagnosed with advanced stages of the disease. Adverse effects, associated with conventional cancer treatment, such as nausea, gastric problems, and weakness are often very unpleasant but tend to be transient and disappear once the treatment is completed. Despite the potential severity of the side effects, conventional treatments are evidence-based and clinically tested, therefore should be considered as the only option to cure the primary disease.

There are five groups of complementary and alternative medicine (CAM) therapies, including alternative medical systems (acupuncture, homeopathic treatment, traditional healers), biologically based therapies, natural products, manipulative and body-based therapies, and mind-body therapies [1]. A broad spectrum of complementary medicine used by patients includes herbs and botanicals, vitamins and minerals, traditional Chinese medicine, homeopathy, and specialized diets [2]. The use of complementary and alternative methods has been found to help reduce anxiety, fatigue, nausea and vomiting, pain, and sleeplessness. It also allows patients to feel more hopeful about the treatment. Many believe that the use of complementary medicine will prolong their life-span and cure their disease [3].

The use of alternative therapies should never be considered the main form of treatment but sometimes can ease the side effects or improve the quality of life by for example reducing pain. National Center for Complementary and Integrative Health [1] defines complementary and alternative medicine (CAM) as a group of diverse health care systems, practices, and products that are not generally considered part of conventional medicine. Complementary methods are used along with medical treatment but are not a part of mainstream medicine, whereas alternative therapies are used instead of standard medical treatment and they have been found either not to work or have been unproven. Even though complementary medicine may help tolerate conventional treatment, it may result in inferior survival due to refusal and/or delays in the start of conventional chemotherapy [4,5,6]. Oncologists are becoming increasingly aware of patients’ use of complementary/alternative medicine (CAM), yet few discuss the use of these therapies with their patients.

It is estimated that 48–88% of patients report the use of complementary and alternative medicine as a part of therapy [3,7,8,9,10]. As not all of the patients share the use of CAM with their doctor, the numbers may be higher. The increasing interest and willingness to use CAM among patients may be due to limitations of conventional treatment. It may be also affected by increased advertising in media or the desire for holistic and or natural treatment. As the incidence of cancer increases and survival time lengthens, the use of CAM is likely to increase. With gaining interest, the population starts to seek information concerning the use of CAM. Usually, it is other patient recommendations, the internet or media, where patients find out about alternative and complementary methods. The above are not reliable sources of information and usually include only the advantages of unconventional treatment and frequently no or limited information about the possible adverse effects.

Some complementary/alternative methods may go along with the chemotherapy treatment but some may cause adverse effects and interfere with the treatment. Any delays or interruptions to the standard treatment may decrease its efficiency and put patients at risk as it gives more time for the cancer cells to spread and grow, potentially reducing relapse rates and time of survival.

Having wondered how frequently our patients use complementary methods of treatment and which methods are the most common, we prepared a questionnaire.

The purpose of this study was to examine the extent of CAM use in a representative sample of cancer patients. This study assessed the prevalence of CAM use among cancer patients treated at comprehensive cancer centers, its safety and sources of knowledge about CAM.

## 2. Materials and Methods

### 2.1. Questionnaire

The questionnaire consisted of 15 questions and was aimed to evaluate patients’ knowledge and experiences with complementary or alternative forms of therapy. The survey was divided into two sections. The first one included patient characteristics such as socioeconomic status, age, education, and general knowledge about CAMs. The second part was dedicated to patients that have declared the use of alternative or complementary therapies and focused on the sources of information about CAM. It also evaluated the frequency and types of therapies used.

The questionnaire combined semi-open-ended questions and closed-ended questions with predefined answers. The patients were instructed to give only one answer, if not otherwise specified in the question. For some questions an optional space was provided to enter a previously undefined answer. The questionnaire is attached as Appendix A.

### 2.2. Survey Sample

Polish-speaking patients, suffering from gynecological malignancies, who were at least 18 years of age and reported for treatment to one of the chemotherapy treatment centers in Szczecin, Poland between 2017 and 2019 were invited to participate in the study. We enrolled 316 consecutive patients who were admitted for treatment in one of the three Oncological Centers in Szczecin which participated in the study. At the time of the survey, all of the patients were undergoing chemotherapy treatment either as a part of treatment for the primary disease or relapse. Patients who suffered from renal insufficiency and hepatic diseases were excluded from the study as any underlining diseases may have limited the potential use of CAM.

After patients signed in to the clinic, doctors and/or nurses introduced the study, describing it as a questionnaire to learn about CAM use and determined patient eligibility. Patients were informed that participation in the study was anonymous and voluntary, and that they could withdraw from the study at any time. Written informed consent was obtained from patients willing to participate in the study. Once the questionnaires were returned, they were coded with a unique identification number to ensure confidentiality. Moreover, we have gathered the information regarding patients’ clinical FIGO (International Federation of Gynecology and Obstetrics) staging, chemotherapy protocol used (platin-based protocol vs. other). As a part of the study, we have conducted a follow-up of patients to determine their overall survival.

The study was approved by the Bioethics Committee of the Pomeranian Medical University in Szczecin KB-0012/58/11. All procedures involving human participation were performed in accordance with the ethical standards of the institutional and/or national research committee, and with the 1964 Declaration of Helsinki and its later amendments or comparable ethical standards.

### 2.3. Sample Size Calculation

Based on our previous clinical experience, we have estimated that approximately 25% of patients treated at our clinic could use CAM additionally to conventional treatment. Using a formula for sample size calculation for qualitative variable:

$$\mathrm{Sample}\;\mathrm{size}=\frac{Z_{1-\frac{\alpha }{2}}^{2}p\left(1-p\right)}{d^{2}}$$

where

*Z*_1−α/2_ is a standard normal variate at 5% type 1 error (*p* < 0.05).
*p* is an expected proportion in population based on previous studies or pilot studies.
Finally, *d* is an absolute error or precision (equal to 5%).

Therefore, we estimated that the study would require at least 289 patients. According to the formula, if the researcher wanted to increase the error and therefore decrease the precision of the study, the denominator would increase and thus the sample size would decrease.

### 2.4. Statistical Analysis

Chi-squared analysis was used to examine the association between CAM use and categorical variables including education, time since the diagnosis, place of residence, decision making, and CAM awareness.

Univariate and multivariate analysis were performed to determine factors associated with CAM use. The concept of odds ratio (OR) was introduced as the ratio between the likelihood of a particular event and the likelihood of that event which did not happen. The odds ratio was used to express the factor of the increase or decrease in the likelihood of a particular event upon a unit change in the independent variable (with fixed values of the remaining independent variables). Statistical significance was considered at *p* < 0.05. We performed overall survival and relapse-free survival analysis using the Kaplan–Meier curves. To compare the relapse survival between groups we used the log rank test. All statistical analyses were performed using the statistical software Statistica (version 10), StatSoft inc.

## 3. Results

### 3.1. Group Characteristics

In total, 316 cancer patients undergoing chemotherapy were enrolled in the study. In the questionnaire we have asked patients’ demographics (age, marital status, place of residence, education level and length of oncological treatment (time since the diagnosis). The group characteristics are listed in Table 1.

Moreover, based on patient’s medical history, we obtained clinical information regarding patients’ clinical staging, and type of chemotherapy regimen used (standard platin-based vs. other.

Based on the gathered data, we performed univariate analysis to check which factors significantly affect CAM use (see Table 2).

In the analysis, we have included parameters such as marital status (married/single), place of residence (city/countryside), education (university level/ other), patients age (above/below median), FIGO clinical staging (FIGO III and IV vs. FIGO I and II), type of chemotherapy protocol used (standard vs. other), and recurrence of the disease (yes/no). The only statistically significant result we obtained in this part of the study was that patients who underwent standard chemotherapy had a 113% increase in risk of using CAM compared to patients who were treated with different chemotherapy protocols. All patients included in the study were females undergoing chemotherapeutic treatment for gynecological malignancy. The mean age of patients was 63.6 years with the youngest aged 38 years and oldest aged 84 years.

As a part of the study, we have also performed multivariant analysis for CAM use taking into consideration the same factors as in the univariant analysis (Table 3). We found that, despite other factors, the type of chemotherapy protocol used in the treatment of patients involved in the study influenced the use of complementary methods of treatment.

**Figure 1:**
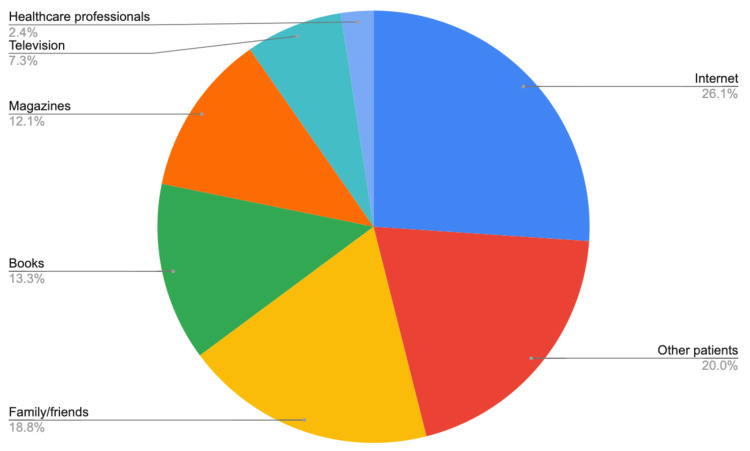
Sources of information regarding CAM methods.

The next part of the questionnaire asked about the sources of information about CAM methods of therapy. The answers chosen by the patients enrolled in our study are presented on Figure 1. As presented on the diagram, most of the patients have conducted their research on the Internet (26.1%). Another popular source of information was an opinion from other patients suffering from similar conditions and equaled 20% of respondents. Other popular choices included patients’ family and friends, books, and magazines. Most of the patients (52.7%) used more than one source of information. Very few patients (2.4%) inquired their doctors or other medical professionals about the types, use, or safety of CAM.

**Figure 2:**
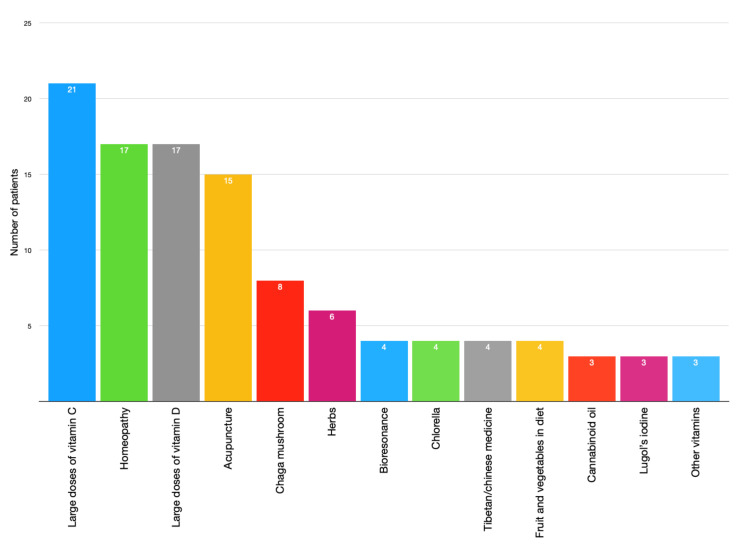
Use of CAM among patients undergoing chemotherapy treatment (methods with less than three users were not included in this figure).

Of the 316 interviewed patients, 27.5% admitted to using any form of CAM therapy. Most of the respondents reported that they used only one method of unconventional therapy (66.7% of users) and 29 respondents used multiple CAMs. The most common type of CAM reported in our questionnaire was the use of high doses of vitamin C, admitted by 24.1% of CAM users. Other common methods included various forms of homeopathy (19.5%), high doses of vitamin D (19.5%), acupuncture (17.2%), Chaga mushroom (9.2%), and various types of herbs (6.9%). The results are demonstrated in Figure 2.

Another correlation demonstrated that patients’ opinion concerning safety of complementary and alternative methods of treatment is associated with its use (*p* = 0.00147). In our study, most of the patients (78.9%) thinking that the use of unconventional methods of treatment is not safe did not use any form of CAM during chemotherapy treatment (Table 4).

Another interesting observation was a correlation demonstrating the influence of patients’ education on their opinion on the possible replacement of traditional therapy with CAM methods. Patients with higher education tended to be more skeptical about this thesis and rarely chose the possibility that CAM can be used instead of a standard treatment (*p* = 0.01198). Our findings are presented in Table 5.

As a part of our research we have also checked the associations between the length of time since the diagnosis and its impact on patients’ opinion on CAM and its use. The time that has passed from patients’ diagnosis did not significantly influence their opinion whether unconventional methods of treatment can replace standard (conventional) cancer treatment (*p* = 0.36052). Moreover, the length of time since the diagnosis did not affect patients’ opinion regarding the need to inform the doctor about the use of unconventional methods of treatment (*p* = 0.52824).

### 3.2. Survival Analysis Using the Kaplan–Meier Curves and COX Regression

We have conducted 42 months of follow-up of patients participating in the study to perform survival analysis. The mean survival was 34.6 months with the minimal survival of 8 months and maximum survival of 42 months. The median overall survival of the studied population was 37 months.

**Figure 3:**
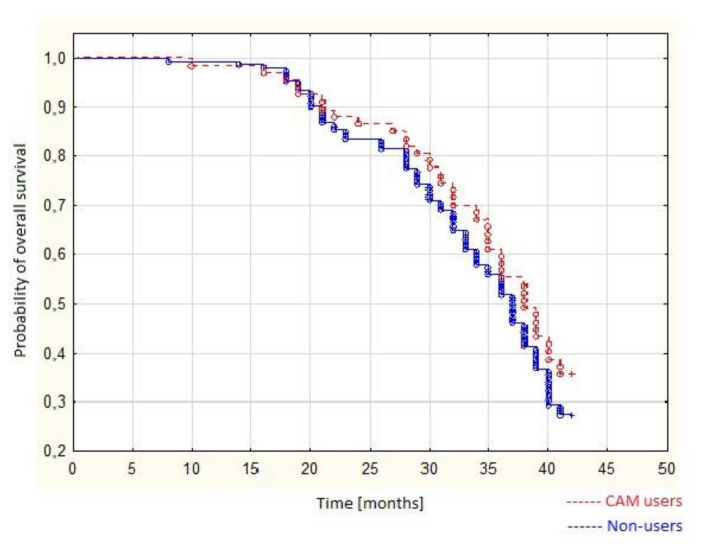
Overall survival of patients.

We performed overall survival analysis using the Kaplan–Meier curves. We conducted survival analysis of patients undergoing chemotherapy treatment with 42 months of follow-up time of our prospective study. The figure below (Figure 3.) demonstrates Kaplan–Meier curves for overall survival of chemotherapeutic patients who used CAM additionally to standard treatment as compared with subjects who did not use any form of CAM. To compare the survival between groups we used the log rank test. We found no statistical difference in overall survival between the groups that used and did not use any form of CAM (*p* = 0.211).

As a part of the study we have also performed a multivariable COX regression model. We found that patients’ age, clinical staging, and recurrence of the primary disease affects patients’ survival, as older patients with higher FIGO staging and cancer recurrence had poorer survival. We found that the use of CAM did not affect patients survival (*p* = 0.066). The results were shown in Table 6.

## 4. Discussion

There are different reasons why patients may want to seek the use of complementary methods in addition to standard cancer treatment. One of them, and probably the most common reason behind its use, is to enhance their quality of life and relieve side effects caused by the treatment such as anxiety, fatigue, pain, nausea, and vomiting [11,12]. Patients often think that the addition of complementary methods including high doses of vitamins and minerals will aid the conventional medicine, combine its effect, and cure cancer. Another common reason was to strengthen patient’s immunity and improve emotional life [3,13,14,15]. Moreover, some of the patients want to take an active role in their treatment, and thus seek additional therapies to be able to choose and decide for themselves [13,16,17,18]. Some want to improve their health and wellness by attending various forms of physical activity, yoga, tai chi, or massages. Over the years, the prevalence of CAM use has seemed to increase. It could either be caused by an actual increase or reflect an increased awareness of CAM among the clinicians [10].

This study was an observational evaluation of CAM use among patients undergoing chemotherapy. The questionnaires used in the study were entirely anonymous. We explored CAMs’ use, patients’ interest, and knowledge, as well as the reason behind its use.

The sample size included 316 patients of white ethnicity and was representative for the group. Our results demonstrate that 87/316 (27.5%) patients used CAM simultaneously with chemotherapeutic treatment. The prevalence is lower when compared with the recently published literature showing that as much as 44–88% of cancer patients used CAM [3].

Use of CAM declared by our patients may be lower than anticipated because of the following: Patients might have been afraid to declare the use of CAM. Physicians rarely ask or advise their patients about possible use and adverse effects of CAM. As reported previously, only about half of the patients who use CAM disclose its use to their health care teams [12,19]. Many patients are afraid to discuss the use of complementary and alternative methods with their doctors, as they worry that they will not approve the use of unconventional treatment methods even along conventional therapy. It is necessary to inform the medical team that one is thinking about a complementary treatment to be sure it will not interfere with the standard medical treatment. It is crucial not to delay or skip regular treatment appointments without discussing it. Patients and doctors need to understand both points of view and discuss and choose safer choices together minding the success of the therapy. Secondly, we proposed some of the answers to the patients; however, we did not include some popular methods such as different herbal therapies, use of green tea, mint tea, ginger, which is very popular in our country. We did not assess any diet changes nor increase of specific food intake (red vegetables, fruits); if patients were willing to declare its use, they had to add it themselves. Furthermore, the wide range of prevalence may be due to different definitions used by various researchers, patients, and the general public. In our study, we have only asked about the pharmacological methods of CAM and acupuncture/acupressure. Previous studies have also included spiritual methods (religion/rituals) and physical activity (for example yoga classes). Other authors included emotional and spiritual forms of CAM. This may be the result of higher percentages of CAM in previous research. As reported by Dy et al. [10] 24.5% of patients have chosen spiritual therapy as a form of CAM. In our study, none of the patients have mentioned the use of spiritual methods (prayer/faith, spiritual healers), psychological (support groups, relaxation techniques, mind and emotion therapy) nor physiological treatment (yoga, massages, touch and movement therapy).

Our study confirms data obtained in previous studies, as vitamins were the most common forms of CAM. Similar to Dy et al. [10], supplements including high doses of Vitamins C and D were the most frequently used by our patients.

High-dose vitamin C usage has been extensively researched in the last decades. Van Gorkom et al. have reported that there was no correlation between vitamin C supplementation and patients’ survival, clinical status, or quality of life [20]. Moreover, Jacobs et al. showed that there was no positive effect of vitamin C usage on antitumor effect or chemotherapy enhancement [21]. On the other hand, O’Leary BR et al. reported a potentially positive effect on lowering the metastatic potential of pancreatic cancer [22], whereas Nauman et al. demonstrated a slight (8.75 months) progression-free survival and overall survival increase in patients using vitamin C [23]. While there are no significant proofs of a positive correlation between vitamin C intake and oncological treatment, there are also no reports indicating lack of safety or harmfulness of such supplementation, even if high dosage is concerned. There is limited literature evaluating vitamin C treatment. Thus, especially considering the popularity of this CAM method, there is a need for placebo-controlled trials in this field.

Other commonly used pharmacological methods included homeopathy, Chaga mushrooms, herbs, and chlorella. Contrary to previous research, our patients did not mention the use of garlic, teas (Green tea, ginseng), nor specific herbs (echinacea, essiac) [9]. Patients often take herbs and supplements, which are advertised to help in liver regeneration and restoration of its function, however the effect is often contrary, and sometimes even worsens liver parameters and function.

Along with the increasing use of CAM and a wide range of substances used as pharmacological forms of CAM, drug to drug interactions, their influence on chemotherapy metabolism, and the occurrence of any adverse effects should be studied. Drug metabolism, pharmacokinetics, and pharmacodynamics should be taken into consideration in toxicity potential of CAM. Alternation of cytochrome CYP 450 and plasma protein binding should be studied when CAM is coadministered with chemotherapy. Interactions between CAM and chemotherapeutics should be determined alone and in combination with the most common CAMs to provide the information to clinicians and their patients.

Polyphenols and catechins present in many teas and herbs have been found to inhibit some of the cytochrome CYP 450 enzymes. They have been seen to cause drug to drug interactions and inhibit some of the chemotherapeutics metabolized by CYP 450 or cause resistance of chemotherapy drugs such as vincristine, vinblastine, taxanes, antracyclines, tamoxifene, and tyrosine kinase inhibitors [9]. On the other hand, some substances are unlikely to cause interactions with most drugs used in chemotherapy, as in the case of medical mushrooms; however, as they nonspecifically activate the immune system, hypersensitivity may be induced. Interactions with homeopathy are also unlikely as due to dilutions of D6 (that is six times 1:10) they no longer contain molecules from the primary extract [24]. Even everyday products such as grapefruit juice, echinacea, garlic, ginseng, and St. John’s wort were found to influence the metabolism of chemotherapeutics and to alter their concentration and clearance [25,26,27].

Among nonpharmacologic methods of CAM, the most frequent were diets including juice fasting and vegetarian diets, as confirmed in our research and use of other practices and alternative practitioners such as homeopathic, osteopathic, or alternative medicine practitioners [10]. The most common was acupuncture, used by 17 patients.

In our research some of the patients used many CAMs simultaneously. In total, 29 patients reported use of more than one CAM method, with the values reaching up to five different methods. The duration of time since the diagnosis was found to increase the chance of using more than two types of CAM by seven times (*p* = 0.00021). Moreover, we found that the length of time since cancer diagnosis, despite other factors, was found to quadruple the chance of using any form of CAM during chemotherapy treatment. Being treated for many years, patients most probably have relapsed and underwent multiple forms of standard treatment. Various protocols and types of treatment (chemotherapy, radiotherapy, target therapy), each carries a specific list of possible side effects associated with them. Having expected some of the side effects in previous lines of treatment, patients may have an increased anxiety and be encouraged to try to relieve them using CAM. Patients who relapsed usually fear that further treatment may not work as well and therefore want to take actions themselves trying every possible method that can help cure cancer.

Regarding the knowledge concerning CAM, patients have searched the information using a variety of methods. The most popular source of information was the internet, chosen by 26% of patients. Other popular sources included information from friends and family or came from other cancer patients. Trends seem to be similar to these previously reported by Molassiotis et al. [28] and Navo et al. [9]. However, in our study, the number of patients receiving information regarding CAM from health care professionals was even lower, and only equal 2.4%. Due to the common use of CAM, medical teams consisting of doctors and nurses should be able to provide reliable information for patients, as common sources may lack quality assessment and therefore cause misinformation [29]. Embracing a more active approach towards the topic of CAM should be considered by medical professionals treating oncological patients. This might cause a shift in sources of information from the less reliable, such as the Internet, to the more reliable, evidence-based and holistic ones. There are potential benefits for the patients—the use of CAM would be safer—with a reduced risk of possible interactions. Eventually, this open-minded approach could also lower the level of patients’ stress and increase compliance with conventional therapy.

Information on CAM in cancer centers and among doctors is often limited and outdated. Despite this, we acknowledge that the use of these therapies is common, and information on contemporary medicine and its possible use are needed for doctors, nurses, and patient educators who should respond to the growing interest among patients.

There are several limitations to the study. Our research included patients undergoing chemotherapy at referral cancer centers, however, we did not differentiate whether the patients were treated for a primary disease or a relapse. Moreover, the questionnaire did not include detailed patients’ characteristics and did not divide patients by sex, age nor type of the primary disease. Although the study included a wide range of patients with diverse tumor types, we did not record their histology, thus we were unable to see if the type of the disease affected the prevalence of CAM use. Furthermore, we did not examine the association between the use of different therapy methods (surgery and/or radiotherapy) in addition to chemotherapy. Johnson et al. [30] discovered an association between cancer staging and the likelihood to use CAM in patients with higher stages of the disease. In our study we found that patients’ clinical staging did not affect the use of CAM. However, we found associations between the type of chemotherapy used (standard vs. other). As the standard chemotherapy protocol, we chose platin-based chemotherapy regimens, which are commonly used in the treatment of various gynecological malignancies. The most common types of side effects associated with this protocol are hair loss, neutropenia, anemia, tiredness, bruising, diarrhea, nausea, vomiting, and constipation. The use of CAM among these patients may be associated with a willingness to lessen the side effects of the chemotherapeutic treatment.

In our study, the time from cancer diagnosis to the date of the survey did not significantly affect patients’ usage of CAM as there were no significant differences between the groups. However, as the questionnaire was administered only once to each patient during their chemotherapeutic treatment, we were unable to see if the duration of the treatment or severity and complexity of side effects have changed patients’ opinions regarding the use of CAM and their safety.

Previous studies have shown the predominance of CAM use among females, married patients, patients of a younger age, those with higher levels of education, and those with private health insurance [19]. In our study, contrary to previous literature [3,7,31], we found no association between CAM use and patients’ place of residence, education, age, nor marital status. As a part of our study, we have also evaluated the impact of CAM use on patient’s survival using Kaplan–Meier and log-rank analysis. We found that the use of any form of complementary form of treatment did not affect patients’ survival, as both CAM users and patients who did not use any form of complementary treatment had similar overall survival times.

Regardless of the limitations, our results can serve as preliminary information concerning the use of CAM in our population. However further research is needed to establish the associations of CAM use among cancer patients undergoing different methods of cancer treatment (for ex. radiotherapy, target therapies). Observational prospective studies including CAMs’ impact on disease-free and overall survival would help define its use in cancer treatment. Clinical trials are essential to help evaluate the potential benefits and risks associated with CAM use. The safety profile and efficacy of CAM methods need to be established.

## 5. Conclusions

Frequent use of CAM among cancer patients is evident. Irrespective of personal opinion, healthcare professionals need to be aware of such use of CAM and be able to provide patients with information and educate them about possible benefits as well as side effects. The concept and understanding of medicine should be broadened to help form an integrated model of medical care combining mainstream standard medical treatment with CAM therapies for which evidence of effectiveness exists [28,32].

In conclusion, our study demonstrates that approximately one-third of cancer patients in Poland have used at least one method of CAM during their treatment. Doctors should be aware of common CAM use and discuss its possible benefits and associated risks with patients. Doctors should possess knowledge about different CAM methods and possible interactions with the drugs used.

## Figures and Tables

**Table 1:** Group characteristics.

	Total Number of Patients *n* = 316 (%)	Complementary and Alternative Method (CAM) Users *n* = 87 (%)	Non-Users *n* = 229 (%)	*p*-Value
Place of Residence				0.570
City <5000 habitants	25 (7.9)	8 (9.2)	17 (7.4)	
City >5000 and <20,000 habitants	49 (15.5)	12 (13.8)	37 (16.2)	
City >20,000 and <100,000 habitants	67 (21.2)	21 (24.1)	46 (20.1)	
City >100,000 and <200,000 habitants	16 (5.1)	2 (2.3)	14 (6.1)	
City >200,000 habitants	104 (32.9)	26 (29.9)	78 (34.1)	
Countryside	55 (17.4)	18 (20.7)	37 (16.2)	
Education				0.671
Primary	27 (8.5)	7 (8.1)	20 (8.7)	
Junior High School	2 (0.6)	0 (0.0)	2 (0.9)	
Vocational	65 (20.6)	16 (18.4)	49 (21.4)	
Senior High School	140 (44.3)	37 (42.5)	103 (45.0)	
Higher	82 (26.0)	27 (31.0)	55 (24.0)	
Oncological Status				0.943
Newly diagnosed (less than 2 years)	154 (48.7)	41 (47.1)	113 (49.3)	
Treated for more than 2 and less than 6 years	86 (27.2)	23 (26.5)	63 (27.5)	
Treated for more than 6 years	69 (21.9)	21 (24.1)	48 (21.0)	
Did not answer	7 (2.2)	2 (2.3)	5 (2.2)	

**Table 2:** Univariate analysis for CAM use.

	CAM USE		
	OR	95% CI	*p* Value
Marital status (Married vs. single)	0.69	0.39–1.22	0.203
Place of residence (city vs. countryside)	0.66	0.36–1.21	0.178
Education (higher vs. other)	1.323	0.70–2.51	0.392
Age (above vs. below median)	0.88	0.50–1.57	0.675
FIGO staging (III and IV vs. I and II)	1.13	0.61–2.07	0.700
Chemotherapy protocol (standard vs. other)	2.33	1.26–4.34	0.007
Recurrence (yes vs. no)	0.74	0.35–1.57	0.433

**Table 3:** Multivariate analysis for CAM use.

	CAM Use		
	OR	95% CI	*p* Value
Marital status (Married vs. single)	0.59	0.33–1.18	0.226
Place of residence (city vs. countryside)	0.84	0.60–1.92	0.371
Education (higher vs. other)	1.41	0.75–1.72	0.032
Age (above vs. below median)	0.93	0.49–2.06	0.711
FIGO staging (III and IV vs. I and II)	1.22	0.76–1.89	0.058
Chemotherapy protocol (standard vs. other)	1.96	1.18–2.43	0.012
Recurrence (yes vs. no)	0.88	0.41–1.38	0.052

**Table 4:** Relationship between CAM use and patients regarding its safety (*p* = 0.00147).

Declared CAM Use	Thinks that Unconventional Methods of Therapy are Safe (in Number of Patients)	
	No	Yes
No	166	29
Yes	35	36

**Table 5:** Association between patients’ education and their opinion on CAM replacing traditional therapy (*p* = 0.01198).

Patients’ Education	Thinks that Alternative Medicine can Replace Traditional Therapy (in Number of Patients)		
	Yes	No	Doesn’t Know
Higher	6	52	24
Senior High School	13	69	57
Vocational	12	23	30
Primary	4	7	16
Junior High School	0	1	1

**Table 6:** Multivariate Cox regression model.

	Multivariate Analysis (Cox Regression Model)		
	HR	95% CI	*p*-Value
CAM use (users vs. non users)	0.98	0.71–1.16	0.066
Age (above vs. below median)	1.12	0.84–1.39	0.023
Marital status (Married vs. single)	0.79	0.63–0.99	0.073
FIGO staging (III and IV vs. I and II)	1.41	1.11–1.71	0.01
Recurrence (yes vs. no)	1.26	1.02–1.39	0.044
Chemotherapy protocol (standard vs. other)	0.91	0.69–1.12	0.071
Education (higher vs. other)	0.78	0.63–0.98	0.103

## Data Availability

The data presented in this study are available on request from the corresponding author. The data are not publicly available due to ethical restrictions.

## Appendix A

Box A1 Survey sample.

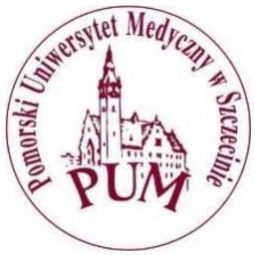

**QUESTIONNAIRE EVALUATING PATIENTS’ APPROACH TO COMPLEMENTARY AND ALTERNATIVE MEDICINE**

Traditional therapy /conventional medical treatment refer to standard, evidence-based forms of cancer therapy (chemotherapy, radiotherapy, surgery), commonly used in the hospitals by doctors and other medical professionals.Unconventional medicine or therapy methods refer to methods that are not a part of standard treatment. Complementary methods are used along with medical treatment but are not a part of mainstream medicine, whereas alternative therapies are used instead of standard medical treatment. The questionnaire is anonymous and voluntary. Its only purpose is scientific evaluation of complementary and alternative medicine use among patients undergoing cancer treatment. The questionnaire consists of semi-open-ended questions and closed-ended questions. In closed-ended questions please select one answer (if not otherwise specified). We kindly ask to give honest and full responses. For the statistical purposes, please specify your Age:

1. How big is your city of residence?
  (a) City < 5.000 habitants
  (b) City >5.000 and <20.000 habitants
  (c) City >20.000 and <100.000 habitants
  (d) City >100.000 and <200.000 habitants
  (e) City > 200.000 habitants
  (f) Countryside
2. Please choose your education level:
  (a) Primary
  (b) Junior high school
  (c) Vocational
  (d) Senior high school
  (e) Higher
3. What is your martial status?
  (a) Single
  (b) Married
4. For how many years have you had the neoplasm? (Please enter the number of years)
5. Do you think that unconventional methods of therapy can be effective and able to help treat a neoplasm?
  (a) Yes
  (b) No
  (c) I don’t know
6. Do you think that unconventional medicine can complement traditional methods of therapy?
  (a) Yes
  (b) No
  (c) I don’t know
7. Do you think that alternative medicine can replace traditional forms of therapy?
  (a) Yes
  (b) No
  (c) I don’t know
8. Do you think that unconventional methods of therapy are safe?
  (a) Yes, they are safe
  (b) No, they are not safe
9. Do you think that a person using unconventional methods of treatment should inform their doctor about such practice?
  (a) Yes
  (b) No
  (c) I don’t know
10. Have you ever used complementary methods of treatment?
  (a) Yes
  (b) No

**Please proceed to this part only if you have answered** “**YES**” **in question number 9**.

11. How often do you use complementary methods of treatment?
  (a) Very rarely
  (b) Occasionally
  (c) Quite often
  (d) Very often
12. Where did you learn about complementary and alternative methods of treatment? (you can select more than one answer)
  (a) Internet
  (b) TV
  (c) Books
  (d) Magazines
  (e) Family/friends
  (f) Other cancer patients
  (g) Medical professionals
13. What are/were your reasons for use of unconventional medicine? (you can choose more than one answer and/or write your own answer)
  (a) No effect of traditional/standard treatment
  (b) To relieve the side effects of standard treatment (chemotherapy/ radiotherapy)
  (c) Positive feedback from other patients
  (d) Influence of friends/family
14. Have you noticed any improvement in your health that you associate with the use of complementary forms of therapy?
  (a) Yes
  (b) No
  (c) Difficult to say
15. What kind of therapy do/did you use? (you can select more than one answer and/or write your own answer)
  (a) Homeopathy
  (b) Chinese/Tibetan medicine
  (c) Bioresonance
  (d) Acupressure/ reflexotherapy
  (e) Acupuncture
  (f) Ozone therapy
  (g) Large doses of Vitamin C
  (h) Large doses of Vitamin D
  (i) Lugol’s iodine
  (j) Silver water
  (k) Chlorella
  (l) Chaga mushrooms
  (m) Other: ……………
